# Combined thoracic medial branch radiofrequency and chemical neurotomy

**DOI:** 10.1016/j.inpm.2021.100002

**Published:** 2021-11-29

**Authors:** Richard Derby, Yakov Vorobeychik, Byron J. Schneider, Jeongeun Lee

**Affiliations:** a Former Chief Medical Officer, Spinal Diagnostics and Treatment Center, Daily City, CA, USA; b Former Clinical Associate Professor, Stanford University, USA; c Penn State Milton S. Hershey Medical Center, Penn State College of Medicine. Department of Anesthesiology & Perioperative Medicine. Hershey, PA, USA; d Interventional Spine and Musculoskeletal Medicine Fellowship Director. Physical Medicine and Rehabilitation, Vanderbilt University Medical Center, USA; e Staff Pharmacist, CVS Health, Daly City, CA, USA; f Former Research Analyst, Spinal Diagnostics and Treatment Center, Daly City, CA, USA

**Keywords:** Chronic pain, Back pain, Radiofrequency, Neurotomy, Thoracic

## Abstract

**Objective:**

Explore the effectiveness of thoracic medial branch neurotomy (MBN) using combined radiofrequency neurotomy and neurolytic lesioning.

**Design:**

A retrospective cohort of consecutive patients with chronic thoracic axial pain treated in a community setting.

**Interventions:**

We included all patients who underwent MBN between 2010 and 2016, selected for MBN primarily based on 70% relief following single or dual diagnostic medial branch blocks. Using 18-gauge cannulas, we placed electrodes and made lesions at the suspected location of the thoracic medial branch based on anatomic knowledge at the time; the thermal lesions were supplemented with 50% dextrose to enhance the lesion radius.

**Measures:**

We defined success as ≥50% relief of their index thoracic pain not returning to baseline for at least six months. Patients not reached for follow-up were considered failures for worst-case analysis.

**Results:**

Twenty-eight patients underwent an initial MBN between 2010 and 2016: Twenty-five of twenty-eight (89%) patients reported ≥70% pain reduction not returning to baseline for six months or longer. Using a worst-case analysis (WCA), patients reported average pain relief of 73% CI (63%,84%) with a mean duration of relief following the initial MBN of 9.9 months CI (6, 13.5). Seventeen of the initial 28 patients had a total of 31 repeat MBNs, 13/17 (76%) having pain relief reinstated after one or more repeat MBNs with an average duration of relief following a first repeat MBNs of 10.9 months CI (6.6,15.2).

**Conclusion:**

Thoracic MBN combined with a mild neurolytic is a potentially effective treatment for thoracic pain in patients selected with positive diagnostic MBB. There were no complications noted. One can reinstate pain relief with repeated MBN in most patients should their symptoms return.

## 1 Introduction

Many peer-reviewed published studies report outcomes following medial branch neurotomy (MBN) in the cervical and lumbar regions; however, few outcome studies exist for thoracic medial branch neurotomy, perhaps best explained by the relatively lower prevalence of thoracic pain. Indeed, it was not until 1995 that Chua and Bogduk performed the first dissections of the thoracic medial branches reporting innervation from the medial branch penetrating the dorsal fascia near the tip of the superior lateral aspect of the thoracic transverse process [1].

Nevertheless, the few early thoracic MBN outcome studies did not place the radiofrequency cannulas nor injected neurolytic agents in the location of the thoracic medial branches as described by Choi & Bogduk [[1], [2], [3]] On the other hand; four recent studies are based on their anatomical placement. A Swedish study in 2018 reported MBN outcomes based on the health-related quality of life EQ-5D instrument. They used a multisite heating location along the cephalad and lateral border of the thoracic transverse process using an 18-gauge radiofrequency needle, reporting thoracic MBN outcomes comparable to cervical and lumbar MBN [4]. A 2020 retrospective cohort of 39 patients selected for thoracic MBN using diagnostic intra-articular injections reported that 46% of the patients achieved ≥50% pain relief at six months post-neurotomy. A more recent retrospective cohort of 39 patients published in 2021 showed a similar 63% of patients who were afforded ≥50% pain relief at six months post thoracic MBN performed by placing an 18 gauge radio-frequency needle tip on the superior lateral tip of the transverse process [2]. Finally, a Scandinavian study by Rohof and Chen reports that most patients (82%) had ≥50% pain reduction at 12 months after bipolar thoracic R.F. neurotomy [3].

Although a previous study compared thoracic radiofrequency MBN to chemical neurotomy using alcohol [4], no study reported its combined use. To help fill this gap, we audited a consecutive series of thoracic MBNs to explore clinical outcomes of the thoracic MBN combined with a neurolytic agent (50% dextrose), categorizing results based on percent relief of subjectively reported index thoracic pain in ten percent increments. We secondary explore whether repeat MBN can reinstate pain relief.

## 2 Methods

We retrospectively reviewed charts of consecutive patients treated at one facility and by the same physician, undergoing one or more thoracic MBNs from January 2010 to January 2016, excluding no patients. We considered all routinely documented medical information collected during procedures and office visits and accessed the clinic evaluations, additional clinical information, procedural reports, and procedural fluoroscopy images via a secure backup server-based database and file system. We obtained IRB approval # IRB00012773 (Eck Institutional Review Board) as a retrospective audit, recognizing that there is no immediate benefit for the selected patients; however, for future patients and providers, study findings may provide better evidence-based patient consent for both MBB and MBN. This research did not receive any specific grant from funding agencies in the public, commercial, or not-for-profit sectors. No authors had any conflict of interest, and the study was conducted according to the Declaration of Helsinki.

Given that this is a retrospective audit of clinical practice, we did not determine *a priori* treatment paradigms*.* However, we observed typical practice patterns at this single site. Patients typically had a documented history and physical examination consistent with posterior element pain, with the physical examination revealing local tenderness over one or more of the thoracic facet joints, extension, and side-bending typically aggravating their symptoms. A minority of patients may have had concomitant pain potentially due to thoracic disc protrusions and pain arising from lumbar or cervical spine regions but were not excluded from this study. Typically, we defined a positive response to diagnostic MBB as at least 70% relief of the index pain. We matriculated two patients with less than 70% pain relief following MBB to MBN, one reporting convincing prolonged relief of index pain on a two-week follow-up, the other wanting the MBN and stating that they would be happy with any degree of relief. In patients under 30 years old, we used an 80% relief threshold to characterize a positive response.

When insurance allowed, in 6/25 subjects, we performed a second confirmatory MBB before their initial MBN, and only if it was again positive did the patient proceed to MBN. We did not repeat MBBs before offering a repeat MBN in patients who had a successful first MBN, but their index pain later returned.

### 2.1 MBB

After a sterile prep and drape, we performed MBB using a 1.5-inch 25–gauge needle placed onto the superior-lateral thoracic transverse process using intermittent A.P. fluoroscopy, followed by the injection of 0.5 ​ml of 0.75% bupivacaine. (Figure A.4). A facility nurse tested patients 45–60 ​min following the MBB recording a VAS (visual analog scale), and in most cases also documented pain during functional patient movements, including flexion, extension, side bending, sitting, standing, and walking. In selected cases, a nurse performed a second evaluation 2 ​h after the patient returned to the ASC after being discharged to self-test. In most post-block evaluations, the treating physician verified the nurses finding. Typically, the patients would be seen in the clinic for follow-up in 2 weeks to discuss the results of the MBB.

### 2.2 MBN

We performed all MBNs in the same ambulatory surgical center operating room using fluoroscopy to guide the needles. We placed either a single or two 18-gauge Teflon-coated needles (NeuroTherm, Wilmington, MA, USA) with a 10 ​mm exposed tips on the superior lateral aspect of the thoracic transverse process. In 30/52 MBNs, we placed two 18 gauged needles side by side to create a bipolar lesion (Figure A.5). After lesioning at 90° for 90 ​s, we injected 0.5 ​ml of 50% dextrose, typically using a separate 25-gauge needle placed on the superior-lateral tip of the transverse process (Figure A.6). In lower thoracic levels and for four later cases, we also included a more proximal lesion using the above “two-needle” technique [[5], [6], [7]] (Figure A.8).

### 2.3 Follow-up

We tabulated the response to MBBs performed before the first neurotomy and dictated follow-up data at the scheduled 6-week follow-up when the patient returned for an MBN follow-up, and at between 6 and 12 months by a clinical coordinator by phone if the information was not available on the patient's chart.

## 3 Results

**Fig. A.1:**
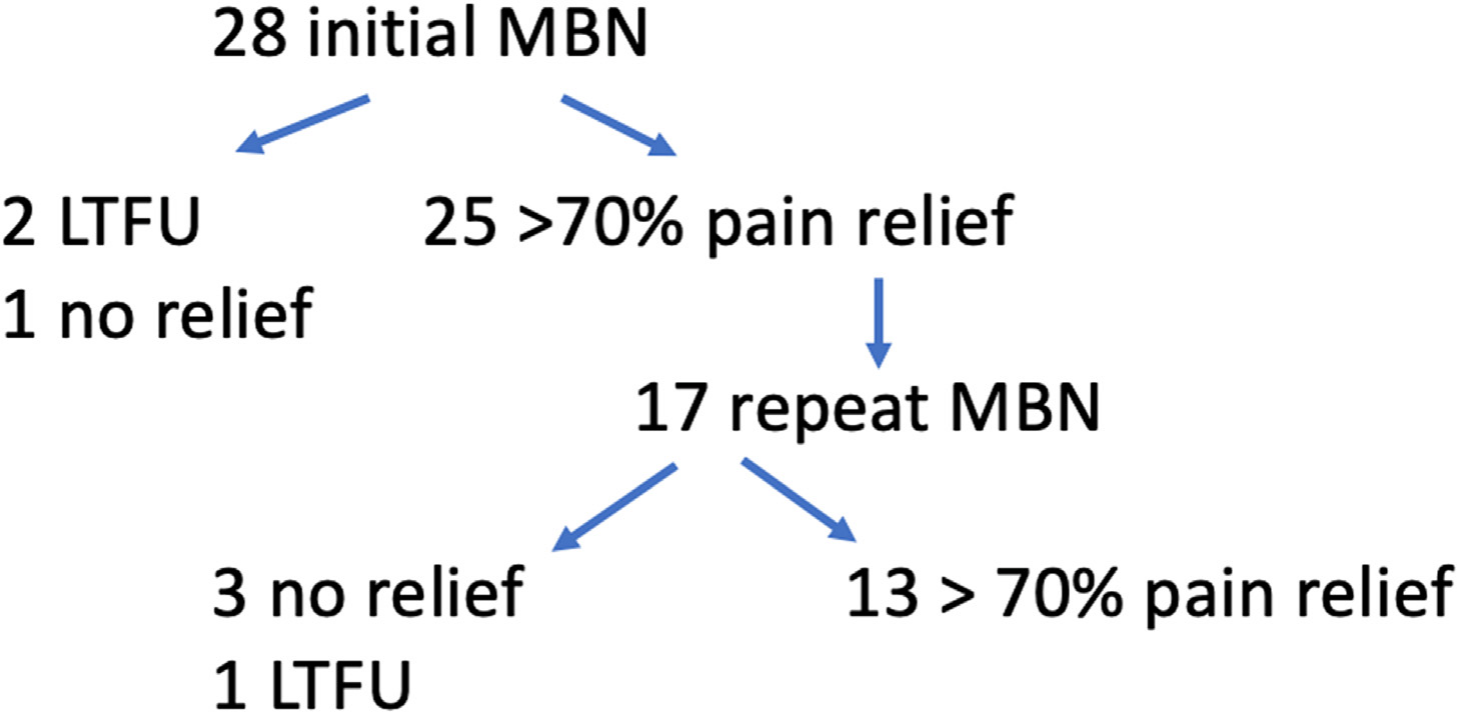
Patient Flow Diagram.

We identified 28 patients who collectively underwent a total of 56 MBN after accounting for repeat MBNs (Figure A.1). Patients proceeding to MBN after a successful MBB had an average decrease in pain following MBBs of 86% S.D. (±12.9). We performed a confirmatory MBBs in 6/25 patients with a positive first block when insurance allowed, all confirmatory MBBs having a positive second block, with an average of 89% S.D. (±14.0) relief of their index.

**Fig. A.2:**
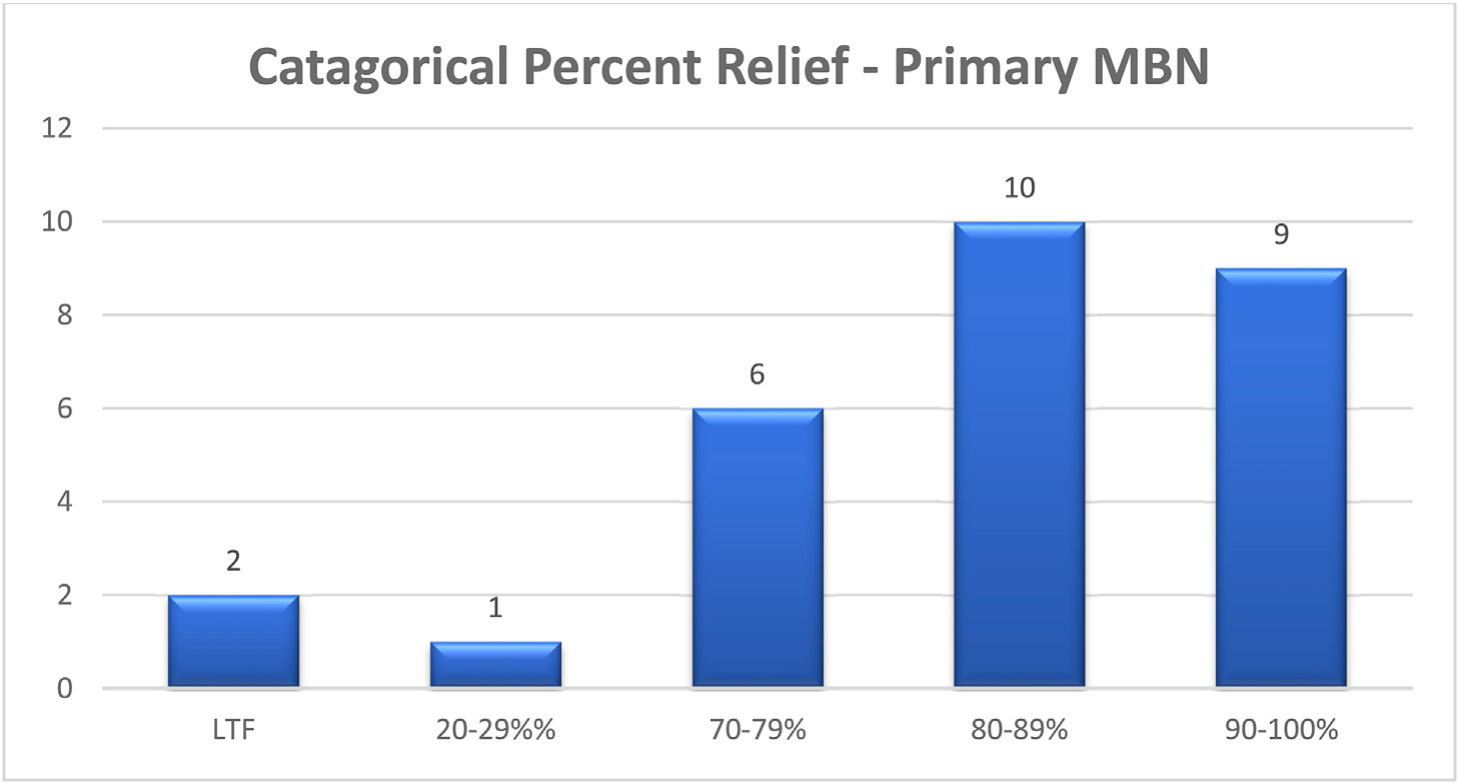
Patient categorical % pain relief at six months or longer post-medial branch neurotomy (MBN)

After their initial MBN, 25/28 (89%) achieved ≥70% relief for six months or longer (Figure A.2). In the best-case analysis (BCA), patients averaged 79% CI (72, 84) relief; worse case-analysis (WCA) - 73% CI (63, 84) (Table A.2). The mean duration of relief following initial MBN in the WCA was 9.1 months CI (6,.9 11.3) (Table A.3). The two patients lost to follow-up after their initial MBN were presumed failures, and we recorded these cases as 0% relief utilizing worst-case data analysis. Thirteen of seventeen (76%) patients returning for repeat MBN had pain relief reinstated with each repeat MBN, with an average duration of relief following the first repeat MBN in WCA 10.9 months CI (6.6,15.2) months (Table A.3).

We lost two patients to follow-up after their first MBN and one patient undergoing a first repeat MBN after a successful first. In our worst-case analyses, we used 0% pain relief for the patients reporting pain returning to baseline before six months. Tables A.2 and A.3 summarize worst-case and best-case analyses.

One patient underwent fusion for a thoracic disc herniation and radicular pain following their first MBN, although their first MBN provided significant pain relief of their axial symptoms. One patient with failed kyphoplasty, severe kyphosis, and osteoporosis had relief of their index thoracic pain over the targeted levels following their first MBN but remaining pain below the levels targeted by MBN. The patient reported 100% relief following repeat MBB but failed to have sustained relief beyond two months after repeat MBN; the failure was most likely technical or due to the other pain sources (Figure A.7). Four patients had continued pain relief at the time of follow-up. There were no complications besides temporary post-procedural pain due to the needle insertions. Procedural pain typically lasted less than a week, although one patient with a supplementary diagnosis of fibromyalgia and on opioids complained of localized pain for a month.

## 4 Discussion

We retrospectively confirm the favorable MBN outcomes reported by four recent MBN studies that similarly selected patients based on MBB relief. Comparatively, we offered MBNs to patients reporting effective pain relief after MBB. We primarily used a 70% cutoff rather than 80% and 50% cutoffs by the Burnham and Speldewinde studies as in our patient population, the 70% cutoff best correlated with the favorable prognostic value of MBBs [8]. Further, we supplemented our radiofrequency MBN with neurolysis using 0.5 ​ml of 50% dextrose.

Doing so and using WCA, 89% of patients reported 70% or more pain relief not returning to baseline for six months or longer, reinstating pain relief in 13/17 patients requesting repeat MBN. The average pain relief lasted 9.1 months after the first MBN and 10.9 months after the first repeat MBN.

We performed MBB and MBN based on the previously established thoracic facet joint innervation coming from the descending branch of the medial branch originating at the superior lateral border of the transverse process [1]. We placed one or two closely spaced 18-gauge radiofrequency needles semi-parallel to about 60° inclination to the thoracic transverse process's superior lateral border, creating a more expansive lesion to help cover variations of the medial branch locations [3,6,7,9]. (Figure A.5, A.6) In the lower thoracic levels, we typically placed the radiofrequency needles at the center to the base of the transverse process (Figure A.8). We injected .5 ​ml of 50% dextrose on the distal upper border of the transverse process to hopefully incorporate medial branches running or exiting midway between the process that may not be within the radio-frequency heat lesion radius (Figure A.6).

Interestingly, Joo et al. reported longer-lasting relief using alcohol than radiofrequency MBN, performing thermal lesions and alcohol injections medially, at the base of the transverse process, close to the facet joint rather than laterally closer to the penetrating medial branch [4]. We supplemented the radiofrequency lesioning with 0.5 ​ml of 50% dextrose that has an osmotic shock effect with evidence showing mild neurolysis at concentrations at or above a 10% [10]; our decision to supplement radiofrequency lesioning with a dextrose solution prompted by our anecdotal use of 6% phenol in glycerin in a patient that had undergone several prior successful neurotomies. The stronger neurolytic did not extend nor improve their pain relief, and the patient complained of a month of localized pain over the injection sites.

**Fig. A.4:**
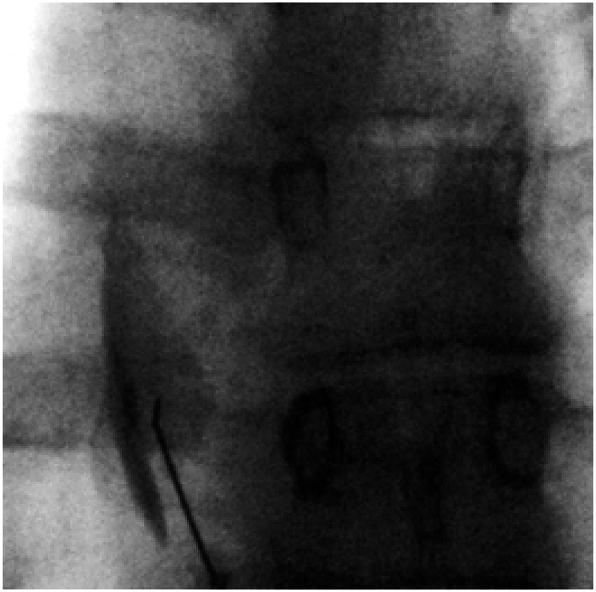
A 1.5-inch twenty-five-gauge needle with a distal bend is on the superior lateral edge of the T6 transverse process. Injection of 0.5 ​ml of contrast examples a typical superior-caudal spread of local anesthetic during MBB or 50% dextrose during MBN

**Fig. A.5:**
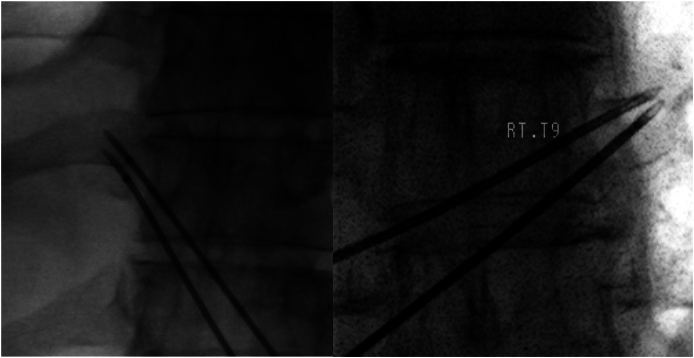
Two examples of a “two-needle” technique Entering the skin approximately 1–2 cm contralateral to the midline at or slightly above or below the caudal aligned endplates, two 18-gauge needles with curved distal tips are separately advanced with the curved tip down until one contacts the base of the transverse process. One then rotates the needle 180° to approximate a more parallel direction to the transverse process and advanced over the transverse process to its superior lateral aspect. Keeping a flat trajectory and not advancing beyond the tip of the transverse process, one can advance the tip over the edge as the more medial needles demonstrate.

**Fig. A.6:**
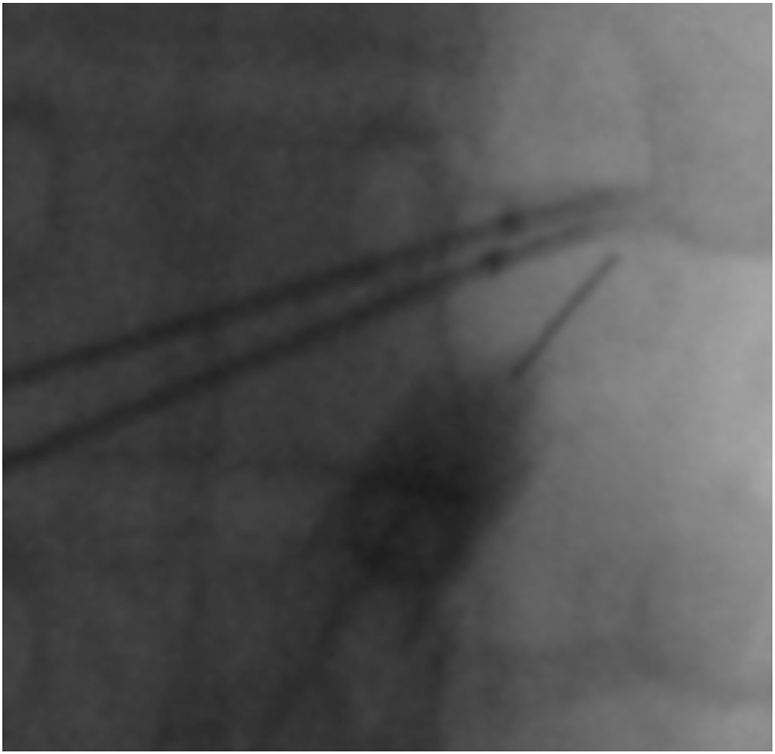
Chemical Neurotomy After radiofrequency lesioning, one places a 1.5-inch 25-gauge needle onto the superior lateral edge of the transverse process and injects 0.5 ml 50% dextrose. Alternatively, one may inject through one of the radiofrequency needles. Note that the radiofrequency needles are positioned slightly above and adjacent to the superior edge of the transverse process to help incorporate the medial branch into the lesion radius. However, it is often challenging to keep two needles stationary, determine their exact location of an osteoporotic T process, and cope with the general veracities of placing two-needle side-by-side.

**Fig. A.7:**
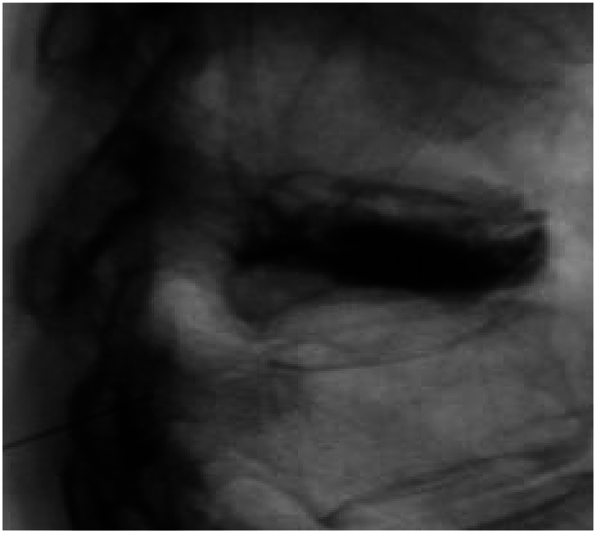
Example of a failed repeat medial branch neurotomy (MBN) The left image is the lateral image of the MBB. After the first MBN above the kyphotic deformity, the patient had relief of her upper index pain but continued to have lower thoracic pain that failed to improve after lower thoracic MBNs. The failure was possibly due to proximal dorsal root entrapment, inadequate nerve ablation, or both.

**Fig. A.8:**
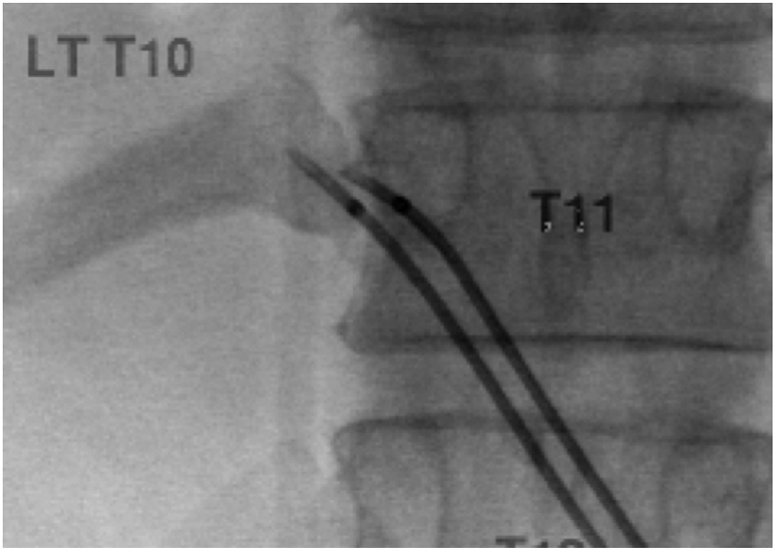
A two-needle technique at the T11 vertebrae shows a more proximal needle placement. We typically used a modified lumbar needle positioning for T10-T12 transitional segments. The patient reported 80% pain relief and continued relief on 38-month follow-up second repeat T7-T10 MBNs

However, one cannot determine if the additional injection of 50% dextrose is of any additional benefit or, indeed, the neurolytic effect of hypertonic dextrose alone would provide a comparative outcome. On the other hand, no prior thoracic studies raised the possibility that the combined use could benignly increase the success rates of radiofrequency lesioning. Indeed, the spread of a neurolytic solution should reach the nociceptive nerve fibers in the intertransverse space (Figure A.4), and, additionally, one could consider multiple site injections.

One must also consider that success or failure of MBNs may not be solely due to denervating the facet joint. The anatomical dissections of Ishizuka et al. [11] found that in over 50% of the cases, the innervation of the thoracic facet joints was provided by a descending branch from the dorsal ramus. Joshi et al. further substantiated their findings in a recent similar but more extensive anatomical dissection, finding in ∼90% of their specimens that the branches innervating the thoracic facet joints originated from the dorsal ramus within or near the neuroforamen; the medial branches most frequently seen between the transverse processes rather than adjacent to the transverse process [12].

Nevertheless, the original studies by Chua et al. report delicate medial branch nerves innervating the joint as the distal medial branch passed medially below the joint [1]. These branches, being only 50 μm in diameter, could be easily missed by Ishizuka's or Joshi's dissections, prompting us in some cases to add proximal lesions to incorporate these superficial articular nerves that may enter from below the joint. Nonetheless, the descending branches from the dorsal ramus described by Ishizuka and Joshi are likely not in a position to be safely incorporated in a radiofrequency burn radius.

One must thus consider that pathology other than structures innervated by the thoracic dorsal ramus nerves may be an additional or primary pain source. Ishizuka et al. opined that the facet joint or its capsule might not cause thoracic axial pain as they found no evidence of facet joint hypertrophy despite an average time of death of 72 years in their cadavers [11]. They postulated that the medial and lateral branches exiting the posterior compartment might become entrapped or compressed by ligaments attached to the transverse processes. Indeed, Joshi et al. recent anatomical studies show the medial branch running within the facia between and parallel to the transverse processes [12]. Thus, one might further opine that chronic medial branch entrapment due to a variety of causes could initiate and maintain a chronic neuropathic pain syndrome, perhaps similar to more advanced cases of notalgia paresthetica purported labeled in 1934 by Astwazaurow as a sensory neuropathy, later circumstantially opined due to a degenerative spinal origin or an entrapment syndrome [13,14]. Similarly, one may entertain the possibility that pain relief is partially a “regenerative” effect of desensitizing and strengthening ligament and tendon attachments to the transverse processes.

Although these opinions merit consideration, medial branch neurotomy techniques that target the medial branch as it exits the posterior compartment between or adjacent to the transverse processes do not eliminate other pain sources from consideration. Pain relief may be due to denervation of the innervated structures such as facet joint, capsule, multifidus muscles, or pain relief may be due to neurolysis alleviating pain caused by posterior compartment nerve entrapments.

So too, any nonrandomized and uncontrolled study's “placebo” effect or patient pain that regresses to a lower mean value by factors other than the treatment is an alternate explanation for subjectively reported pain relief. However, a robust placebo effect typically averages 50% or less and not the 70% or more pain relief reported by our patient cohort using a worst-case scenario. Still, a randomized control trial would likely produce a control group with significant overlap to the treatment group.

Further, the study is a retrospective audit without a control group, we did not record functional outcomes, and patients subjectively reported their percent relief to the treating physician, his staff, or both; deficiencies that may artificially increase subjectively reported relief percentage and duration. In addition, several patients had continuing pain from either cervical or lumbar sources that were not their index pain but influenced both their total pain relief and functional status.

Despite these limitations that predispose to over-optimistic expectations, our outcome data is consistent with prior studies. Provided one performs thoracic MBNs using a comparative technique, we conservatively feel that our data justifies a patient consent that, depending on the case details, could include a reasonable expectation of a 60% or greater chance of clinically significant pain relief for approximately six months. Even for complex cases, such as adjacent segment fusion pain, continuing pain following kyphoplasty, and thoracic kyphotic deformities (Table A1), one could consider thoracic MBN an option, although not a cure, for patients, not candidates for, or before, surgical reconstruction or spinal cord stimulation.

## 5 Conclusion

In summary, thoracic MBN in our study, selected by convincing relief following MBB, is a low morbidity procedure with comparable outcomes to lumbar MBN [8]. Consistent with MBN results in the cervical and lumbar spine, one can usually sustain pain relief by repeating the MBN.

## Conflict of interest statement

The following authors are on the board of directors for the Spine Intervention Society: Richard Derby, Yakov Vorobeychik, Byron Schneider.

The following authors are on the editorial staff of Interventional Pain Medicine: Richard Derby, Yakov Vorobeychik, Byron Schneider.

No other potential competing interests are known to exist.

## Figures and Tables

**Table A.1:** Patient demographic and clinical characteristics. Kyphoplasty (Kypho.), Percocet, Fentanyl, Morphine (Morphine Eq)

Gender		Pre MBN-Numerical Pain	
Male	14	Median pain	7
Female	14	Interquartile Range	7.0–8.0
**Age**		Range	6.0–9.0
Median	52.5	**Nerves Treated (1**st **MBN)**	
Interquartile	24	T2	1
Range	25–81	T3	2
**Injury**		T4	6
Trauma		T5	10
MVA	5	T6	15
Falling	6	T7	23
Altercation	3	T8	21
Lift-Push	3	T9	21
Repetitive	3	T10	11
Unknown	5	T11	6
**Diagnosis**		Bilateral	13
Comp Fracture	7		
Kyphosis	6		
Adj Fusion	4		
DDD	8		
Fibromyalgia	2		
Myofascial	5		
Disc	2		
Post-Kypho.	4		
**Narcotics**	11		
Hydrocodone	5		
Morphine Eq	6		

**Table A.2:** Worst Case (WCA) and Best Case Analysis (BCA) of Percent Pain Relief at ​≥ ​6 ​mo follow-up. Std Dev(Standard Deviation), CI (Confidence Interval)

	Primary MBN	
	WCA	BCA
No Patients	28	26
Avg % Relief	73.4%	78.7%
Std Dev	26.5.3%	17.6%
95% CI	10.3%	6.8.0%

**Fig. A.3:**
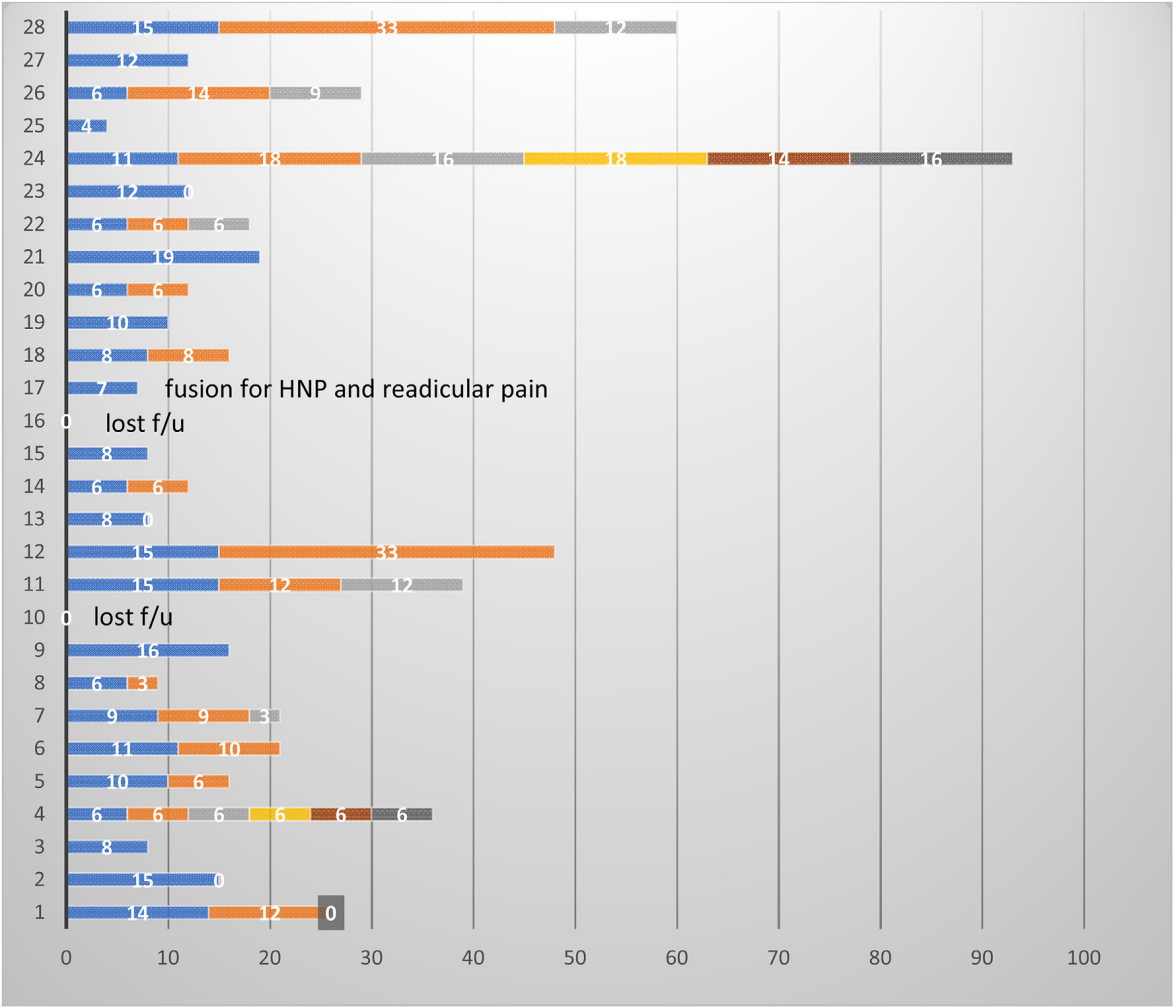
Individual Patient MBN Durations Legend: Each bar represents one or more consecutive MBNs performed on one patient, each different color representing a separate MBN. The x-axis numbers are months.

**Table A.3:** Duration of pain relief following medial branch neurotomy (MBN)s Worst Case (WCA) and Best Case Analysis (BCA)

	Duration 1st MBN		Duration 1st Repeat MBN	
	WCA	BCA	WCA	BCA
No MBNs	28	26	18	17
Avg months	9.1 ​mo.	9.8 ​mo.	10.9 ​mo.	11.6mo.
Std Dev	5.7 ​mo.	5.0 ​mo.	9.4 ​mo.	9.3 ​mo.
95% CI	2.2 ​mo.	2.2 ​mo.	4.3 ​mo.	4.4 ​mo.

Legend: The text box provides the average and standard deviation of a best case and worse case analysis after the first MBN. The repeat MBN is average, and the standard deviation of the first repeat MBN.
